# Impact of alternative reimbursement strategies in the new cooperative medical scheme on caesarean delivery rates: a mixed-method study in rural China

**DOI:** 10.1186/1472-6963-12-217

**Published:** 2012-07-24

**Authors:** Kun Huang, Fangbiao Tao, Lennart Bogg, Shenglan Tang

**Affiliations:** 1 School of Public Health, Anhui Medical University, Hefei City, Anhui Province, PR China; 2 Division of Global Health (IHCAR), Department of Public Health Sciences, Karolinska Institutet, Stockholm, Sweden; 3 Duke Global Health Institute, Duke University, Durham, NC 27705, USA

**Keywords:** Caesarean delivery, Health insurance, Maternal health services, Reimbursement mechanisms, Rural China

## Abstract

**Background:**

The rate of caesarean delivery (CD) in rural China has been rapidly increasing in recent decades. Due to the exorbitant costs associated with CD, paying for this expensive procedure is often a great challenge for the majority of rural families. Since 2003, the Chinese government has re-established the New Cooperative Medical Scheme (NCMS), aimed to improve the access of essential healthcare to rural residents and reduce financial burden owing to high out of pocket payments. This paper seeks to test the hypothesis that NCMS may provide service users and providers with financial incentives to select CD. It also assesses the effect of different health insurance reimbursement strategies of NCMS on CD rates in rural China.

**Methods:**

Mixed quantitative and qualitative methods were adopted for data collection. Two cross-sectional household surveys were conducted with women having babies delivered in 2006 and 2009; 2326 and 1515 women, respectively, from the study sites were interviewed using structured questionnaires, to collect demographic and socio-economic data, maternal and child care characteristics and health-related expenditures. Focus group discussions (FGDs) and in-depth key informant interviews (KIIs) were undertaken with policy makers, health managers, providers and mothers to understand their perceptions of the influence of NCMS on the choices of delivery mode.

**Results:**

The CD rates in the two study counties were 46.0 percent and 64.7 percent in 2006, increasing to 63.6 percent and 82.1 percent, respectively, in 2009. The study found that decisions on the selection of CD largely came from the pregnant women. Logistic regression analysis, after adjusting for socio-economic, maternal and fetal characteristics, did not indicate a significant effect of either proportional reimbursement or fixed amount reimbursement on the choice of CD for both study years. Interviews with stakeholders reflected that different reimbursable rates for CD and vaginal deliveries did not have a significant effect on controlling the rising CD rate in the study countries.

**Conclusion:**

NCMS reimbursement strategies adopted in the study counties of China did not have a significant effect on the selection of CD for baby deliveries. The rapid rise of the CD rates of rural China has remained a serious issue. Other effective measures, such as health education to increase awareness of mothers' knowledge, and improving training of health staff in evidence-based delivery care, maybe could do more to promote rational baby delivery in rural China.

## Background

Caesarean delivery (CD) is needed to prevent or treat life-threatening maternal or foetal complications in an estimated 5–15 percent of pregnancies [1,2]. During the past few decades, however, increasing CD rates have been a growing concern in many countries [3-5]. Of sixty middle-and high-income countries reviewed in a recent study, the majority (62 percent) of the countries had average CD rates above 15 percent [6]. The World Health Organization (WHO) Global Survey on Maternal and Perinatal Health reported that in 2007–08 the overall facility-based CD rate was estimated to be 27.3 percent in Asia with the highest estimate for China at 46.2 percent [7]. The CD rate in China has increased rapidly during past decades [8], increasing twice as fast in the rural areas as in the urban areas [9].

Socio-economic, institutional, financial and professional determinants related to both service provider and user can be attributed to the rapidly increasing CD rates [10-13]. Caesarean delivery requires vast health care resources, and its practices have been associated with responsiveness to a variety of economic forces, including source of payment, malpractice liability, and financial incentives [14]. A review of country-level financing mechanisms for maternal health in a number of countries found that direct out-of-pocket payments reduced utilization of maternal health services and that pooled prepaid schemes (e.g. health insurance) could help increase access [15]. Studies found that increased levels of health care funding were linked to improved access to maternal and child health services [16-18]. A higher share of the government funding in the total health care expenditure was associated with increased utilization of skilled birth attendants and a higher CD rate in many developing countries [19]. But there are other contradictory reports about the effect of health insurance on CD rates. For instance, in Brazil, where the CD rates weigh heavily on the national health insurance fund, equal pay for all types of delivery was instituted to control CD rates from 1980, but this led to only a temporary halting of the increase in number of babies delivered by caesarean delivery. The effect of other Brazilian government initiatives to reduce caesarean births, such as the limitation of CD rates established by the Brazilian Unified Health System (SUS)**,** the funding agency for the national health insurance scheme, and **“**Pact for the Reduction of Caesarean Sections”, were also short-lived [20]. In the United States, it is estimated that an increase of USD 100 in the absolute differential between Caesarean and vaginal delivery reimbursement increased the CD rate in the Medicaid population by 0.7 percentage points [21]. In Taiwan, the National Health Insurance (NHI) found that reducing the reimbursement differences between Caesarean and vaginal deliveries was likely to have only limited or no effect on the demand for Caesarean deliveries [22]. However, in Shanghai, which is ethnically and culturally related to Taiwan, the high Caesarean delivery rate increased with the introduction of health insurance [23].

In China, government funding for maternal health care of rural residents has been negligible until the reintroduction of the New Cooperative Medical Scheme (NCMS) in 2003. During the last few years, the central government in China has paid growing attention to the need for reducing financial barriers in accessing essential care for the rural population. The NCMS was introduced to address problems of inequity in financing of, and access to, quality health care [24,25]. It is designed with cost-sharing between central government, local government and the participants. The Chinese Ministry of Health is responsible for developing principles and guidelines of the NCMS, while local health authorities take major responsibility for the development of detailed operational plans and policies as well as the implementation of the schemes. Due to different fiscal statuses of local governments in China, their financial contributions to NCMS vary a great deal, which has resulted in variations of the service benefits packages provided.

While NCMS may have helped meet unmet needs of healthcare in rural China [26], its impact on the choice of caesarean delivery in rural populations has yet to be unveiled. NCMS in rural China, irrefutably the largest health insurance system, in the world covers a huge population. Therefore, it will have important policy, design and operational implications for the emerging movement of universal health coverage in developing countries. Evaluation of the effects of NCMS policies on caesarean deliveries, especially under the condition of dramatially increasing CD rates, would provide an opportunity to examine how best the insurance reimbursement should be designed to ensure both equity and efficiency for service provision. Since the NCMS aims to enhance access to health services, we hypothesised that NCMS may increase the utilization of caesarean deliveries. In addition, we also want to understand the effects of alternative reimbursement strategies of NCMS on selection of caesarean deliveries in rural China.

## Methods

The data presented in this paper was generated as part of a broader international collaborative research project entitled “Structural hinders to and promoters of good maternal care in rural China (CHIMACA)”. Ethical approval was obtained from the Biomedicine Ethical Committee in Anhui Medical University (Approval No. 2007002).

### Settings

Two counties of Anhui Province in eastern China were selected, using the following criteria: 1) Local government interest in the project and willingness to participate; 2) Prior NCMS implementation in study counties; 3) No other ongoing maternal health intervention program within study counties during the study period. It was agreed with the county leaders that any publications emanating from the study would not disclose the identity of the participating counties, so that acronyms of the study counties are used in this paper. In County FC, all 18 townships were selected as study sites. In County XC, 12 townships were selected according to different geographical accessibility.

Table 1 shows the baseline information for the two counties FC and XC. The average income per capita in the two counties was higher than the nationwide average net income per capita in rural areas, which was reported to be 3587 Chinese yuan in 2006. The income in FC was 1.4 folds higher than that in XC. County XC was larger with relatively more hospitals able to provide CD. Although fewer obstetricians able to perform a CS were observed in XC, population catchment per hospital able to provide CS was also smaller in XC.

**Table 1:** Baseline demographic and socio economic information for two counties in Anhui province

**Indicators**	**FC county**	**XC county**
**Population and economic**		
Total population*	462,244	842,265
Reproductive age women:15–49 years (percent of total)	121,483 (26.3)	220,187 (26.1)
Average GDP (yuan)^#^	11678	10265
Average income per capita (yuan)^#^	5338	3926
**Facilities and staff**		
Number of hospitals	23	44
Number of hospitals able to provide CD	11	43
Population served per hospital able to provide CD	42,022	19,588
Number of general medical doctors	360	513
Number of obstetricians	82	95
Obstetricians per 1000 women of reproductive age	0.67	0.43
Number of midwives	29	56
Midwives per 1000 women of reproductive age	0.24	0.25

Source: FC county health bureau and XC county health bureau.

* FC county statistics bureau and XC county statistics board.

^#^ The average exchange rate (Yuan per US Dollar) in 2006 was 7.973, 2010 rate is 6.830, calculated based on period average published on website of the People’s Bank of China (http://www.pbc.gov.cn/).

NCMS was introduced in 2005 in County FC. From the beginning, it was a proportional reimbursement system with different deductible amounts for different levels of hospital care. Both vaginal and caesarean deliveries are treated as hospitalized cases. Women were reimbursed at different proportions of actual costs depending on the level of services used (e.g. county or township). Table 2 shows the percentage of reimbursement for the different level of hospital care in County FC. Beginning in 2007, fixed reimbursement was adopted to cap the reimbursement ceiling for caesarean delivery. Regardless of hospital care level at which women delivered, they can get 150 Chinese yuan for vaginal delivery and 600 Chinese yuan for caesarean delivery, making payments predictable for households participating in NCMS.

**Table 2:** Reimbursement proportion of hospital costs in county FC

**Cost in hospitals (Yuan)**	**Reimbursement proportion**		
	**Township hospitals**	**County hospitals**	**Regional hospitals**
201-2000	45%	80% of the township hospitals	60% of the township hospitals
2001-5000	50%		
5001-10000	60%		
10001-20000	70%		
above 20001	90%		

In County XC, NCMS was launched in 2007, providing a fixed reimbursement for the cost of baby delivery. The reimbursement was made at the same rate for both vaginal and caesarean delivery. Irrespective of the level of hospital being utilized, the mothers received the reimbursement of 300 Chinese yuan for either vaginal or caesarean delivery.

Baseline health system study findings performed by our research team revealed the general procedure for maternal health care in the two counties. Rural women usually had their first antenatal visit within three months of pregnancy in their residential township hospital and made appointments for regular antenatal care. Typically, women would choose the same provider for antenatal care and for delivery, except under the following circumstances: when referred to high-level hospitals due to pregnancy-related complications, when re-located to other places during delivery, or when they wanted to choose a better hospital to have childbirth deliveries. Referral was required for both antenatal and delivery care. If the doctor could not manage the complications during pregnancy or childbirth, women would be referred to a higher-level institution; for instance, from township level to county level, or even from county level to city level. The reimbursement for the costs of delivery were calculated based on the local NCMS regulations for childbirth, and the costs of complications were calculated according to the local health insurance policies applied to inpatient services [26].

Due to the limited professional staff at grass-roots hospitals (for example, township hospitals), there was no clear distinction between obstetricians and midwives. Doctors were responsible for both vaginal delivery and caesarean delivery. In FC County, caesarean delivery was not performed in all township hospitals. A shortage of skilled staff prevented some township hospitals from conducting CD. There were clinical guidelines on caesarean section at the provincial level. Three specific indications—fetal distress, dystocia, and previous caesarean section accounted for most caesarean sections. Other relative indicators, such as large-for-date infant and circular umbilical cord had different weights in decision-making of delivery mode. There were no clear supervision mechanisms put in place to monitor the use of clinical indicators for the selection of CD, and it relied much on the physicians’ and/or women’ preference [26].

### Data collection

The study used a combination of quantitative and qualitative methodology to collect data in the study counties on the operation of the NCMS and its effect on maternal choice of delivery mode. Quantitative methods included two household health interview surveys conducted in December 2006 and January 2009, respectively. Qualitative studies mainly consisted of a number of focus group discussions (FGDs) and in-depth key informant interviews (KIIs).

The two household surveys aimed to collect data on provision and utilization of maternal health services. In the 2006 survey, one-third of townships of each county were randomly chosen. All villages in these townships were included in the sample. Within these villages, women who had given birth between January 2005 and December 2006 were recruited as participants. In the 2009 survey, all selected 30 townships were put into the sample and in each township, one-third of villages were randomly chosen. In these villages, women who had deliveries from March to December 2008 were recruited as participants. Senior researchers from Anhui Medical University organized a training workshop in which young researchers were able to receive appropriate training on interview skills and contents of the survey questionnaires, as well as other relevant issues. Following training, a pilot study was conducted in one or two villages of County FC. Survey questionnaires were revised based on the results from the pilot studies. Following the survey revision, selected women were interviewed by using the structured questionnaire, which included general demographic information, socioeconomic status, general medical and obstetric history and utilization of maternal health services in the index pregnancy. Income data for 2006 and 2008, total costs, NCMS reimbursement and household out-of-pocket payment for vaginal delivery and caesarean delivery were collected from the two surveys.

As for the focus group discussions and in-depth key informant interviews, topic guidelines were used to understand changes in the NCMS policies including reimbursement regulation, and effects of NCMS on health providers and service users. The study participants included government officials responsible for maternal health care and NCMS at county level, health managers and providers at township level, as well as health users. A total of 13 FGDs and 7 KIIs were conducted by university faculty members, research/teaching assistants, and graduate students of Anhui Medical University. With the permission of each participant, all interviews and discussions were taped and transcribed.

### Statistical analysis

Questionnaire coding and data entry were performed with EpiData 3.0. Statistical analysis was carried out with SPSS 16.0. The caesarean delivery rates in the two selected counties and the changes between the two repeated cross-sectional household surveys and the associated characteristics were reported using descriptive statistics. The determinants of caesarean delivery were analyzed using logistic regression analysis. Key demographic and socio-economic variables that may be associated with the choice of caesarean delivery were identified in the questionnaire. The primary dependent variable was caesarean delivery during the women’s latest pregnancy. Based on literature review and practice, key independent variables in the analysis were set as maternal characteristics, fetal characteristics and household economic characteristics. Maternal characteristics included maternal age, maternal and husband’s education (total education years), previous adverse pregnancy outcome (abortion, fetal death or still birth), systematic antenatal care during pregnancy (first antenatal checkup within first trimester of pregnancy and subsequent five checkups during second and third trimester of pregnancy), and number of ultrasonic examinations during the pregnancy. Fetal characteristics were preterm baby, male baby, firstborn and big baby, defined as birth weight over four kilograms. Economic indicators consisted of delivery place, annual household income per capita, classified as (1) ≤3000 Chinese yuan, (2) 3001–7499 Chinese yuan, (3) ≥7500 Chinese yuan, and women’s participation in NCMS. All variables were dichotomous variables, except for total education years and number of ultrasonic examination during pregnancy, which were continuous.

Data derived from the 2006 and 2009 surveys were used to compare changes in the relationships between the above determinants and caesarean delivery. To obtain an accurate estimate of NCMS parameters, other determinants known to possibly influence the mode of delivery, such as maternal and fetal characteristics, and demographic factors were controlled.

The ‘frame-work approach’ was employed to analyze the qualitative data, using a common analytical framework based on key concepts investigated in the topic guides and themes emerging from an initial reading of the transcripts [27]. All qualitative data was coded, sorted, and classified using Maxqda2 software. Triangulation of participants and researchers enabled crosschecking of the data, allowing for different viewpoints.

## Results

A total of 2326 and 1515 women completed the questionnaires in two household surveys undertaken in 2006 and 2009, respectively.

### NCMS and socio-economic characteristics of the household survey participants

Socio-economic characteristics of the survey participants are presented in Table 3. Over 85 percent of the study participants were members of NCMS in 2009 in County FC, while 81.9 percent of the participants in County XC were NCMS members. According to the surveys, those not participating in NCMS were not covered by other health insurance schemes. The average self-reported household incomes per capita in 2009 improved significantly by 51.6 percent and 43.0 percent, respectively in FC county and XC county, in comparison with the average incomes reported in the 2006 survey. Average costs for both vaginal delivery and caesarean delivery increased in the two study counties, with statistical significance in County FC.

**Table 3:** Maternal health care and economic characteristics from 2006 and 2009 survey

**Characteristic**	**County FC**		**County XC**	
	**Original NCMS (2006) n = 1227**	**Current NCMS (2009) n = 1099**	**Pre-NCMS (2006) n = 896**	**Post-NCMS (2009) n = 619**
**Maternal characteristics**				
Maternal age (mean ± SD)	27.20 ± 4.60	26.95 ± 4.57	28.29 ± 5.26	28.12 ± 5.48
Maternal education years (mean ± SD)	6.58 ± 2.97	7.58 ± 2.70**	6.13 ± 3.22	7.25 ± 2.92**
Husband education years (mean ± SD)	7.80 ± 2.26	8.45 ± 2.25**	7.53 ± 2.29	8.21 ± 2.46**
Previous adverse pregnant outcome (n/percent)	65/5.3	284/31.7**	98/8.9	184/29.7**
Systematic antenatal care (n/percent)	400/32.6	400/44.6**	430/39.1	342/55.3**
Numbers of ultrasonic examination (mean ± SD)	2.09 ± 1.39	2.94 ± 1.48**	3.05 ± 1.56	3.81 ± 1.75**
**Fetal characteristics**				
First baby (n/percent)	901/73.6	634/70.8	688/62.7	396/64.0
Big baby (n/percent)	139/11.3	118/13.2	83/7.6	35/5.7
Male baby (n/percent)	630/51.3	501/55.9*	604/55.0	312/50.4
Preterm baby (n/percent)	80/6.5	88/9.8**	60/5.5	46/7.4
**Economic characteristics**				
Household annual income per capita (mean ± SD)	5035.3 ± 3940.2	7631.1 ± 7900.3**	5134.6 ± 3514.3	7340.1 ± 5521.8
Participation in NCMS (n/percent)	938/76.4	765/85.4**	-	507/81.9
Average costs for hospital delivery (yuan) (mean ± SD)				
Vaginal delivery	1050.3 ± 709.6	1424.5 ± 902.8**	1346.1 ± 665.2	1526.0 ± 721.6
Caesarean delivery	2796.2 ± 1494.5	3544.9 ± 2465.8**	2433.6 ± 1220.0	2510.6 ± 1404.9
Total costs for hospital delivery (yuan) (mean ± SD)^#^				
Vaginal delivery	1030.8 ± 731.9	1463.8 ± 812.1**	-	1574.0 ± 868.4
Caesarean delivery	2755.4 ± 1564.8	3541.0 ± 2623.7**	-	2488.8 ± 1433.5
NCMS reimbursement (yuan) (mean ± SD)^#^				
Vaginal delivery	133.4 ± 159.4	286.8 ± 311.5**	-	251.1 ± 155.0
Caesarean delivery	575.0 ± 322.5	581.8 ± 394.3**	-	266.7 ± 177.1
Household out-of-pocket payment (yuan) (mean ± SD)^#^				
Vaginal delivery	896.6 ± 671.6	1177.0 ± 975.4**	-	1322.9 ± 884.8
Caesarean delivery	2180.3 ± 1600.1	2959.2 ± 2505.4**	-	2222.0 ± 1439.5

* P<0.05, ** P<0.01;

^#^ only for those who participated in NCMS.

For those who participated in NCMS, the total costs, NCMS reimbursement and household out-of-pocket payment for vaginal delivery and caesarean delivery were described in Table 3. In FC County, in 2006, the NCMS covered 12.9 percent and 20.8 percent of total costs for vaginal delivery and CD, respectively, and a higher proportion was found in the CD reimbursement. In 2009, it covered 19.5 percent and 16.4 percent for vaginal delivery and CD, respectively, and a higher proportion was found in the reimbursement for vaginal delivery. In XC County in 2009, the NCMS covered 15.9 percent and 10.7 percent, respectively, for vaginal delivery and CD. Regarding actual out-of-pocket payment in FC county, CD costs accounted for 43.3 percent and 38.8 percent of household annual income per capita, respectively, in 2006 and 2009. And in XC county, CD costs accounted for 30.3 percent of household annual income per capita in 2009 after reimbursement.

### Changes in maternal health care

The two surveys indicated that the women and their husband’s education level increased in both counties. Proportions of women with previous adverse pregnancy outcomes and systematic antenatal care notably increased (Table 3). As for the use of antenatal care, 90.5 percent and 97.5 percent of the women from County FC used ultrasound in 2006 and 2009, respectively. In County XC, the use was 95.9 percent and 98.5 percent respectively in the two study years. As for hospital-based delivery rate, the 2006 survey showed 99.4 percent (1220/1227) and 99.0 percent (1088/1099) in County FC and County XC, respectively. Based on the data from the 2009 survey, the rate was 100 percent in both counties.

### Changes in Caesarean delivery rates between the two surveys

The two surveys witnessed an increase in caesarean delivery rate in both study counties from 2006 to 2009, as shown in Table 4. In 2006, the CD rates were 46.0 percent and 64.7 percent in Counties FC and XC, respectively, rising to 63.6 percent and 82.1 percent in 2009. The rates increased by 17.6 and 17.4 percentage points in County FC and County XC, respectively. Caesarean delivery rates at the county level hospitals were usually higher than that of the township level hospitals in County FC, as reflected by both surveys. Surprisingly, however, CD rate of County XC in 2009 was higher at the township hospitals than at the county level hospitals. In both counties, the increase of CD rates was greater in the township hospitals than in the county hospitals. In County FC, the CD rate increased by 9.3 percentage points at the county hospitals and 23.6 at the township hospitals. In County XC, the increase was by 4.4 and 20.1 percentage points in the county hospitals and the township hospitals, respectively.

**Table 4:** Caesarean delivery rate in different level hospitals in two counties from 2006 and 2009 survey

**County**	**Year**	**N**	**County level**		**Township level**		**Total**	
			**n**	**percent**	**n**	**percent**	**n**	**percent**
County FC	2006	1227	339	59.9	225	34.6	564	46.0
	2009	896	305	69.2	265	58.2	570	63.6
County XC	2006	1099	258	69.4	441	64.2	711*	64.7
	2009	619	96	73.8	412	84.3	508	82.1

(* The other 12 women had caesarean dellivery in private institutions).

The women responded in the survey that they were the ones who made the decision to have a caesarean delivery in a majority of the cases. The decisions made by women themselves increased from 46.8 percent and 53.6 percent, in County FC and County XC in 2006, to 50.9 percent and 67.5 percent in 2009, respectively, see Table 5. In the questionnaire, women were also asked of the reason for their choice of CD. The women indicated fear of pain (the percent of cases was 43.2 percent and 43.4 percent in 2006 and 2009 in FC, respectively and 43.3 percent and 38.2 percent in XC, respectively) and beliefs that caesarean delivery was safer for both mother and baby (the percent of cases was 46.2 percent and 44.8 percent in 2006 and 2009 in FC, 39.4 percent and 54.2 percent in XC, respectively) as the most common reasons for the choice.

**Table 5:** Primary decision-makers for caesarean delivery in two counties from 2006 and 2009 survey

**County**	**Year**	**N of caesarean deliveries**	**Primary decision makers (n/percent)**		
			**Doctors**	**Women**	**Others**
County A	2006	564	270/47.9	264/46.8	30/5.3
	2009	570	207/36.3	290/50.9	73/12.8
County B	2006	711	289/40.6	381/53.6	41/5.8
	2009	508	145/28.5	343/67.5	20/3.9

### Effects of NCMS on determining caesarean delivery

In County FC, we compared the determinants of caesarean deliveries in terms of changes in NCMS reimbursement policies. After adjusting for maternal, fetal and other economic factors, neither the original proportional reimbursement nor the current fixed reimbursement strategy was found to be associated with a higher probability of caesarean delivery in 2006 or 2009. Table 6 also shows that number of ultrasound examinations during the pregnancy period and level of hospital used for delivery were found to have significant and positive coefficients, supporting that women with more ultrasound examinations during their pregnancy or the women who delivered at the county hospitals were more likely to have caesarean delivery.

**Table 6:** Logistic regression analyses of caesarean delivery – pre/original NCMS, post/current NCMS in two counties

**Characteristic**	**County FC**		**County XC**	
	**Original NCMS (2006) OR/95%CI (n = 1220)**	**Current NCMS (2009) OR/95%CI (n = 1098)**	**Pre-NCMS (2006) OR/95%CI (n = 896)**	**Post-NCMS (2009) OR/95%CI (n = 619)**
**Maternal characteristics**				
Maternal age (24-31y as control)				
≤ 23y	0.768(0.551-1.072)	0.729(0.516-1.030)	0.809(0.558-1.171)	0.730(0.418-1.275)
≥ 32y	1.499(0.983-2.285)	1.090(0.686-1.732)	1.512(1.043-2.192)*	1.263(0.643-2.480)
Maternal education years^#^	1.000(0.948-1.056)	1.072(1.005-1.143)*	1.032(0.980-1.085)	1.110(1.013-1.218)*
Husband education years^#^	1.043(0.975-1.114)	1.009(0.937-1.086)	1.031(0.963-1.104)	0.987(0.886-1.099)
Previous adverse pregnant outcome^@^	1.324(0.757-2.315)	1.753(1.272-2.414)**	1.618(0.967-2.708)	1.317(0.806-2.151)
Systematic antenatal care^@^	0.797(0.599-1.059)	1.269(0.938-1.717)	1.345(0.989-1.828)	0.727(0.456-1.159)
Numbers of ultrasound examination^#^	1.309(1.179-1.452)**	1.164(1.043-1.298)**	1.146(1.036-1.267)**	1.183(1.019-1.374)*
County hospitals	2.378(1.828-3.094)**	1.535(1.154-2.043)**	1.079(0.807-1.441)	0.460(0.283-0.749)**
**Fetal characteristics**^@^				
First baby	1.581(1.071-2.334)*	1.055(0.696-1.600)	1.203(0.840-1.722)	1.338(0.690-2.595)
Big baby	1.146(0.763-1.722)	1.304(0.846-2.009)	0.817(0.488-1.367)	0.650(0.285-1.480)
Male baby	1.012(0.782-1.310)	1.046(0.787-1.391)	1.297(0.984-1.709)	0.892(0.581-1.368)
Preterm baby	0.653 (0.381-1.120)	0.920(0.571-1.482)	0.826(0.461-1.479)	1.010(0.459-2.226)
**Economic characteristics**				
Household annual income per capita (=3000yuan ascontrol)				
3001-7499yuan.	0.960(0.715-1.290)	0.985(0.652-1.490)	1.154(0.842-1.581)	0.868(0.456-1.653)
≥7500yuan	1.303(0.892-1.903)	0.979(0.630-1.521)	1.112(0.736-1.681)	0.690(0.355-1.344)
NCMS participation				
Proportional reimbursement	1.189(0.871-1.624)	-	-	-
Fixed reimbursement	-	0.931(0.613-1.415)	-	1.007(0.560-1.812)

* P<0.05, ** P<0.01; ^#^ continuous variables; ^@^ dichotomous variables.

In County XC, we also compared the factors influencing the choice of caesarean delivery before and after the NCMS implementation. After adjusting for other variables, the NCMS implementation had no significant effect on caesarean delivery. Number of ultrasound examinations during the pregnancy was found to have significant and positive association with caesarean delivery. In 2009, the deliveries at the township hospital were found to be associated with a higher probability of caesarean delivery. See Table 6.

### Stakeholders’ perceptions of the effect of NCMS on the choice of caesarean delivery

Almost all policy-makers, health providers and women thought that different patterns of NCMS reimbursement had no effect on choice of delivery hospital. Women were more concerned about the quality of care provided. Service providers who are highly-skilled and have good equipment are of importance. Generally speaking, county hospitals are often better resourced in terms of staff, facilities and infrastructure. They were reluctant to choose hospitals which cannot conduct caesarean deliveries because of worry that such a service may be required and referral services may not be available immediately. The following direct quote portrays the various perceptions of stakeholders.

"If I had high risk factors, and doctors at the township hospitals could not deal with the delivery, even if I could get more reimbursement from NCMS, I would risk my life and the baby’s life. So usually we consider more of skills and equipment, not of money" (Woman, County FC).

In-depth key informant interviews with hospital managers reported that changing proportional reimbursement to fixed reimbursement for CD, which was expected to control CD rates, did not have significant impact on the choice of delivery mode. Below is what the health managers reported on the issue.

"Proportional reimbursement may have some effect on caesarean delivery rate. Caesarean delivery would cost 2000 yuan and women could get nearly 1000 yuan back. They could have caesarean delivery with only a little payment. Now the fixed 600 yuan RMB did not bring any effect on women’s choice of delivery mode"(Manager, County FC).

""I thought NCMS ought to give reimbursement to women per capita, not by delivery mode. Increasing caesarean delivery rate is caused mainly by social factors, not real medical factors. Anyway, it was our ideal thoughts to decrease caesarean deliveries. Now, there’s no signs of a decreasing rate at all, there still show an increasing trend year by year" (Director of MCH station, county XC)."

""A few women are likely to choose vaginal delivery for economic factors, but the policy will not necessarily (result in) a decrease caesarean delivery rate" (Manager, County XC)."

In addition, the qualitative studies also explored what factors the women would value most in their decision making in terms of delivery mode. The findings reveal that rather than an economic factor, women are more concerned with a safe delivery for both themselves and their baby.

""No matter how much money it will take, I will choose caesarean delivery" (Woman, county XC)."

""Having a baby and getting reimbursement are totally different things. We must consider our physical condition and doctor’s advice. Before delivery, it seems that no one will choose a delivery mode just considering how much money they will get back"(Woman, County XC)."

"“The income level has now become higher and higher. Generally speaking, most families can afford the costs of operational delivery. Reimbursement is not the main factor for choosing a caesarean delivery” (Women, FC)."

## Discussion

Data from the two repeated household surveys clearly show a significant increasing CD rate in the rural areas of China over the past years, particularly at the township hospitals of both counties. Additionally, the surveys indicate that the impact of NCMS reimbursement policies might not be significant on the choice of baby delivery mode. The results do not support our original hypothesis. It seems that the high rates of caesarean delivery cannot solely be attributed to the NCMS implementation. Socio-economic factors, combined with people’s perceptions might also be associated with such a level of CD rates in the study rural counties of China.

Although WHO officially withdrew its previous recommendation of a 15% CD rates in June 2010 considering that there was no empirical evidence for an optimum percentage, the high CD rate reported in our study, however, is far beyond the professionally accepted level. It may have serious implications for cost and quality of healthcare. Introduction of NCMS in rural China may have improved access of rural residents to healthcare services in recent years. Owing to moral hazards, the insured are likely to use healthcare services, even if not absolutely necessary. Fee-for-service, a main provider payment method widely used in China, has also resulted in cost escalation and excessive medicalization, and created a supply-induced demand for the use of more expensive procedures, such as caesarean delivery [28,29]. Bogg et al. had used the quantitative demographic, administrative and accounts data in the study counties and found an increasing trend of CD rate in the provider payment mechanisms in the NCMS. But it was just a presumption and the authors concluded that the design of NCMS needed to be studied to rein in the unhealthy increase in rural CD rates [29]. In our study, policy makers have recognized that proportional reimbursements could be a factor driving the demand for caesarean deliveries to a rapid increasing level. In County FC, fixed reimbursement rates for vaginal and caesarean delivery were therefore introduced. Although the proportion of reimbursement for CD decreased in FC and a fixed reimbursement was introduced in XC, neither of the strategies appeared to have significant influence on the CD utilization. One study examined the impact of an insurance reform that equalized fees for vaginal and caesarean delivery and only found a modest 0.7 percent reduction in Caesarean delivery rates, which appeared to indicate little intensity response to fee changes [30]. A study by Hueston and his colleagues in the US showed that Health Maintenance Organizations were associated with lower rates of CD. To decrease caesarean delivery rates by one percent, however, HMO penetration for white women would have to increase by an average of 20 percent. It was therefore unlikely that changes in insurance status alone would have a significant impact on caesarean delivery rates among these populations [31]. The studies suggest that the choice of delivery mode is influenced by a wide variety of factors and that understanding the local context is vital to any effort made to influence maternal choice of delivery mode. Other authors believe that when women were classified into those who had received reimbursement from NCMS and those who had not, it was found that health insurance “coverage” was associated with total caesarean deliveries [32]. The possible explanation was that the overall economic development level and individual income levels in our study area were more significantly important than the financial subsidies provided by NCMS to the decision of selecting of CD. As presented in Table 3, the annual household income level in both study areas has risen rapidly over the study period. The main reasons are complex. The following factors might be associated with the income increases: i) Policy changes—Chinese farmers no longer need to pay agricultural tax and receive nine years of free compulsory education since mid-2005, giving the rural households more money to spend on other things, and ii) Remittances—an increasing number of farmers have left the countryside to get better paying jobs in cities, sending the money back to their households. According to the State Statistical Bureau of China, the average annual income per capita in rural areas in China was reported to be 3587 Chinese yuan in 2006 and 4761 Chinese yuan in 2008 [33-35].

In addition, although the fixed reimbursement rate provides some financial protection for households, it would only go a small way to reduce the overall financial barrier and is not the sole consideration for choosing caesarean delivery. Findings from the surveys and qualitative analysis also suggested that the costs of caesarean deliveries were an economic burden for the average rural family. However, many households seemed to be willing to pay, as their income levels have increased steadily these years. It could be argued that the fixed reimbursement rate for CD and other services, as a regressive financial mechanism, is affecting equity in access to healthcare and may increase higher risk of “catastrophic” payment for low-income households.

It is apparent that health insurance is only one of several factors that may result in increasing and excessive application of CD in China and other countries. Many factors, apart from the economic aspects, influence the choice of caesarean delivery. For example, women’s fear of pain, concern for the safety of mother and infant, and both health providers’ and users’ low confidence of vaginal delivery play a role in the decision-making process [26]. In our study, it was found that the proportion of women who made the CD decisions had increased in both counties between the year of 2006 and 2009, more than half caesarean deliveries being decided by women themselves. The study’s finding was mainly based on the women’s recall in the household survey. They could clearly speak out the CD decision-maker, while their recall on the medical reasons for CD selection might not be highly reliable. So in the text, therefore, we just dare to use the description “women made the CD decisions themselves” instead of “CD on maternal request”. The definition of “CD on maternal request” are various in the medical literatures. In some researches, it was defined as “primary elective cesarean delivery in the absence of a medical or obstetric indication” [36,37]. However, MacDorman MF et al. argued that this concept did not take into account the role of obstetric care providers [38]. And in their report of the First National U.S. Survey of women’s childbearing experiences, for a woman to have a primary cesarean on maternal request, she needed to meet two criteria: (1) had CD for no medical reason and (2) made the decision for herself before labor [39]. DeClercq and colleagues adopted the term “no indicated risk” (NIR), ie. full-term, singleton, vertex presentation births with no medical risk factors or complications of labor or delivery. They used the United States birth certificate data (including comprehensive population-based nature of CD data but no actual measure of maternal request) and found that the overall rate of primary cesareans in NIR mothers was 6.9% in 2003 [40,41]. Although there are widespread discussions in the literature about maternal request cesareans, most studies had mentioned “the absence of medical indications”. While in the current household survey, due to the poor reliability of women’s recall on medical reasons, as well as the limitations in the medical records in grass-rooted hospitals, we could only simply use the term “women made the CD decision themselves”, whether they had medical reasons or not.

Many caesarean deliveries were performed based on women’s decision with insufficient information of the risks and health consequences of alternative modes of delivery. The lack of knowledge regarding quality services is likely to be a contributory factor in the common perception by women that instrumental examination and surgical intervention at birth is indicative of quality [15,42]. Additionally, results from the qualitative study performed by our research team revealed that request for an auspicious birth date, provider-induced demand due to provider payment mechanisms and revenue-related bonus payments, as well as defensive medical care all contributed for the women’s decision of CD [43]. Since there are few systematic data available on cesarean delivery by maternal request, further research is recommended into the measurement and interpretation of CD on maternal request as a means of further understanding underlying determinants of caesarean delivery. And particularly in our study, the possible medical indications of caesarean delivery underlying maternal decision and the complex and nuanced interactions between mothers and health providers in decision-making must be considered.

Our study also found that the use of ultrasound examination during the pregnancy was associated with the CD choice. It was further explored in another published article by our research team [44]. Ultrasound screening is widely used for confirmation of viability and gestational age, identification of multiple pregnancy and screening for fetal anomalies [45]. But no clear benefit of a substantive outcome, such as reduction of perinatal mortality, has yet to be determined from the routine use of ultrasound [46,47]. The availability and widespread use of ultrasound scanning could be “a marker for a type of patient who prefers medical intervention,” whereby doctors may be inclined to offer caesarean delivery even without clinic indications [48].

In the previous qualitative health system study, it was reported that young physicians who had just graduated from medical school and initiated their hospital work would have very few opportunities to attend to a vaginal birth due to an increase in caesarean deliveries. In conjunction with the improvements of caesarean delivery technology, they would be much more skilled in operational deliveries and the consequent low confidence in normal birth attendant [43]. Moreover, there was an increasing irrational social climate of upward comparison in rural areas, which regarded caesarean delivery as the symbol of high economic level and more family’s concern on puerperal [43]. These may partially explain the increase in caesarean deliveries over time in the two rural areas.

It was also observed that delivery in county hospitals was associated with CD in County FC, but the opposite was the case in County XC. Local health system factors might explain some of the differences. In XC, almost every hospital at both township and county level could perform caesarean deliveries, however, most were performed in township hospitals. In contrast, only half of hospitals could provide CD in FC County. Only a few township hospitals had the capacity to conduct caesarean deliveries, thus more women would go to county health institutions. Additionally, although there were fewer obstetricians per 1000 women of reproductive age in XC than in FC, the CD rate was still higher in XC. Therefore, quality of care and efficiency should be closely monitored, since the health system did not implement strong controls on quality, volumes of services and medical interventions prescribed.

A strength of this study was a combined use of quantitative and qualitative methods. We found it extremely meaningful to assess the causality of observed associations and to explore the perceptions and beliefs surrounding CD among health care providers in the health system via qualitative methodology, in order to give us supplemental and supportive illustration for the quantitative results. Of course, there are obvious limitations in the implementation of this study. First, the analyses of caesarean delivery should have been divided by whether the delivery was planned before the labor (elective) or not (emergency), and by whether it was performed before the onset of labor (antepartum) or during labor (intra-partum). For the constraints, we had to rely on the self-reported data in two household surveys, which could suffer from recall bias. Second, financing mechanisms have a number of potential effects on both service users and providers that need to be disentangled [25]. Providers may also play a role in driving up costs by encouraging expensive procedures covered by insurance, including caesarean delivery. In this study, the effect of NCMS on providers’ behavior was not thoroughly analyzed as it was not an original study aim. Although, for the most part the mothers themselves decided to choose caesarean delivery, it cannot be excluded that the recommendations and advice by the doctors may also be very important in the decision process. Last, our study can be viewed as a case study in rural eastern China. In our study, we just explored the effect of alternative reimbursement strategies on CS in rural areas with extremely high CS rates. The effects of NCMS on CD in less developed areas of China where rural households do not have much income levels may be different to what we have observed in Auhui Province.

## Conclusion

This study can be viewed as a case study to explore if there is a role for NCMS in the overall decision-making for caesarean delivery. The results show an alarming increase in caesarean delivery rates in both study counties in the study period, most of which was determined by women's request. The findings from the study conclude that neither proportional, nor fixed reimbursement strategy introduced by NCMS had an influence on choice of delivery mode. While there is no perfect provider payment method, health systems must develop an appropriate approach that best suits local contexts. Our findings suggest that it is formidably difficult to control caesarean delivery solely relying on financial reimbursement offered by health insurance schemes. More effective health interventions, such as evidence-based health education programs for both service users and health professional staff, need to be developed to reduce caesarean delivery rates to a healthier level recommended by the WHO.

## Competing interests

The authors declare that they have no competing interests.

## Authors' contributions

FT, ST and KH participated in the design of the study. KH and LB involved in data collection and performed statistical analysis. KH, ST, LB and FT drafted the manuscript. All authors read, commented on and approved the final manuscript.

## Pre-publication history

The pre-publication history for this paper can be accessed here:

http://www.biomedcentral.com/1472-6963/12/217/prepub
